# Sperm quality but not relatedness predicts sperm competition success in threespine sticklebacks (*Gasterosteus aculeatus*)

**DOI:** 10.1186/s12862-015-0353-x

**Published:** 2015-04-26

**Authors:** Marion Mehlis, Anna K Rahn, Theo C M Bakker

**Affiliations:** Institute for Evolutionary Biology and Ecology, University of Bonn, An der Immenburg 1, D-53121 Bonn, Germany

**Keywords:** Cryptic female choice, Fish, Paternity, Inbreeding avoidance, Inbreeding depression, Mate choice, Sexual selection, Sperm-egg interaction

## Abstract

**Background:**

Mating between close relatives often leads to a reduction of an individual’s fitness, due to an increased expression of deleterious alleles. Thus, in many animal taxa pre- as well as postcopulatory inbreeding avoidance mechanisms have evolved. An increased risk of inbreeding and hence a loss of genetic variation may occur during founder events as in most cases only few individuals establish a new population. The threespine stickleback (*Gasterosteus aculeatus*) is a small externally fertilizing fish species subject to strong sperm competition. Sticklebacks inhabit both marine and freshwater environments and anadromous populations have repeatedly established new genetically less diverse freshwater populations. Previous studies showed that anadromous sticklebacks strongly suffer from inbreeding depression and when given the choice females prefer to mate with unrelated males.

**Results:**

The present study aimed to address whether there exists a postcopulatory inbreeding avoidance mechanism solely based on sperm-egg interactions in sperm competition experiments. We used F1 individuals that originated either from a large, genetically heterogeneous anadromous population or from a small, genetically less diverse freshwater population. For each population, eggs of two different females were *in vitro* fertilized by the same two males’ sperm in a paired study design. In the main experiment one male was the female’s full-sib brother and in the control experiment all individuals were unrelated. The results revealed that fertilization success was independent of relatedness in both populations suggesting a general lack of a postcopulatory inbreeding avoidance mechanism. Instead, male quality (i.e. sperm morphology) predicted paternity success during competitive fertilization trials.

**Conclusion:**

In sticklebacks, there is no evidence for postcopulatory inbreeding avoidance. Sperm morphology predicted paternity instead, thus sperm quality traits are under strong sexual selection, presumably driven by the high risk of sperm competition under natural conditions.

**Electronic supplementary material:**

The online version of this article (doi:10.1186/s12862-015-0353-x) contains supplementary material, which is available to authorized users.

## Background

In many animal species a reduction of heterozygosity due to inbreeding often results in detrimental consequences for an individual’s fitness (termed “inbreeding depression”, [1]), for example by depressing a male’s sperm competitiveness ([2], but see [3,4]). Beyond that inbreeding may also affect population performance (see [5] for details) and especially small populations with low genetic diversity face an increased risk of becoming extinct (see [6-9] and citations therein).

Precopulatory inbreeding avoidance mechanisms, such as sex-biased dispersal, delayed maturation, mate choice or extra-pair copulations, are well studied (see [10] for an overview). In species in which relatives are likely to encounter each other during adulthood, the ability to recognize kin is a prerequisite to avoid incestuous matings, which was described for various taxa [11-14]. However, if it is not possible to avoid incestuous matings, postcopulatory inbreeding avoidance mechanisms may have been alternatively developed. In some internally fertilizing species, for example, multiply-mated females avoided the negative costs of inbreeding by cryptically favoring the sperm of unrelated males [15-24], but see [25-29]. Postcopulatory preferences for genetically similar mating partners have been documented in the Arctic charr (*Salvelinus alpinus*) [30], the Atlantic salmon (*Salmo salar*) [31] and in the Peron’s tree frog (*Litoria peronii*) [32]. These studies stress that a high possibility of hybridization in their study species accounts for the observed results [30-32]. However, it remains noteworthy that sometimes mating with a close relative is actively preferred and may even increase an individual’s fitness [33-36]. This is in accordance with recent theory, as incestuous matings provide a possibility to spread genes identical by descent [37].

The threespine stickleback (*Gasterosteus aculeatus*) is a small externally fertilizing fish species that inhabits marine, brackish as well as freshwater habitats [38]. Postglacially, anadromous populations have frequently established new freshwater populations, presumably consisting of only a few founder individuals (see [39] for details and [40] for an example of recent colonization events in Switzerland). Due to founder effects and limited population size freshwater populations often show a reduced genetic diversity (e.g. [41,42]), increasing their probability of becoming extinct [43]. This follows the more general pattern that the allelic diversity among fishes is higher in marine populations as compared to freshwater populations (see [44]).

Recent studies on the effects of inbreeding on life history traits exclusively examined stickleback individuals from a large, genetically heterogeneous population from Texel, the Netherlands [41], revealing that they are prone to inbreeding depression. In detail, incestuous matings lowered fertilization success, hatching rate and survival of both juveniles and adults [45]. In addition, at adult stage inbred individuals had more asymmetric pectoral fins [46] and showed an altered shoaling behavior toward kin [47]. Moreover, there is strong evidence for a precopulatory inbreeding avoidance mechanism in this population as in choice experiments, female sticklebacks rejected their brothers as mating partners [48,49], and inbred females had a stronger preference for symmetrical males than outbred females [50]. However, inbreeding did not affect adult males’ breeding coloration or testis and sperm traits [45,51]. This might be best explained by the fact that it is difficult to detect the negative consequences of inbreeding, when homozygous individuals suffer from lethal mutations early in life and as a result a low number of inbred individuals reaches the reproductive phase (see [45,51] for details).

In general, the frequency of sneaking (i.e. the stealing of fertilizations) and thus the risk of sperm competition is known to be high in sticklebacks (e.g. [52]) so that precopulatory inbreeding avoidance (see [48,49]) might not always be effective. Besides, given the severe consequences of incestuous matings (e.g. [45]) the present study aimed to investigate whether there is a postcopulatory inbreeding avoidance mechanism solely based on sperm-egg interactions in sperm competition experiments. Therefore, we used stickleback individuals originating from the same large anadromous population as described before [41] and from a small resident freshwater population (Euskirchen, Germany). Generally, we expected different selective constraints for individuals originating from a small, genetically less diverse compared to a large, genetically heterogeneous population as supported by a recent theoretical study (see [53]). In detail, a postcopulatory inbreeding avoidance mechanism might be less relevant in small populations due to purging of deleterious alleles after repeated inbreeding (e.g. [54,55]). Alternatively, inbreeding is known to enhance the extinction risk in small genetically less diverse populations (see [56]), suggesting that there might be a stronger selection for a postcopulatory inbreeding avoidance mechanism (e.g. [19,57]).

Apart from genetic compatibility [58], sperm morphology, such as sperm size, is known to be a good proxy of reproductive performance in several animal species (reviewed in [59,60]). However, throughout the literature the relationship between sperm size and fertilization success at the intraspecific level is inconsistent: negative (e.g. [61,62]), positive (e.g. [63,64]) or no (e.g. [65,66]) relationships have been reported. Based on the fact that there is huge between- and within-male variation in sperm morphology in the threespine stickleback (see [67]), we additionally addressed the variation of sperm size in relation to fertilization success in the present study. In externally fertilizing species, fertilization rate is not confounded by characteristics of the female reproductive tract.

## Methods

### Ethics

The study conforms to the Association for the Study of Animal Behaviour Guidelines for the use of animals in research as well as to the legal requirements of Germany. We had the permission to catch the parental generation of the F1 sticklebacks used in this study at the Euskirchen field site (local forestry department, Euskirchen, Germany). In addition, the parental generation of the F1 sticklebacks used in this study from the anadromous population (Texel, the Netherlands) was purchased from a commercial fisherman, who has the permission to catch the fish. No further licenses were needed.

### Experimental subjects

Test fish from the small, genetically less diverse freshwater population used in this study originated from the F1 generation of randomly crossed wild-caught fish (2007, Euskirchen, Germany). Parents were only used once to avoid pseudoreplication. Offspring hatched between September 2 and October 11, 2007. During development, all fish were housed in an air-conditioned room under standardized laboratory short-day conditions (17 ± 1°C, day length 8L:16D). Juveniles were fed with *Artemia* nauplii and later on defrosted red mosquito larvae (*Chironomus* spec.). To stimulate reproductive behavior the light-regime was changed to long-day summer conditions (17 ± 1°C, day length 16L:8D) on June 6, 2008. The standardized *in vitro* fertilization trials were conducted between June 19 and July 29, 2008.

Test fish from the large, genetically heterogeneous anadromous population (Texel, the Netherlands) used in this study were also the F1 generation of randomly crossed wild-caught fish (bred in 2008). Breeding and rearing conditions of these individuals followed a similar standardized protocol as for the freshwater population, which is described in detail in Mehlis & Bakker [68]. *In vitro* fertilization trials took place between June 29 and July 23, 2009.

To validate that the F1 freshwater fish used in this study were genetically less diverse in comparison to the F1 anadromous fish we genotyped 34 randomly chosen unrelated individuals (17 from each population) at nine microsatellite loci ([41,69], see also Additional file 1). The results showed that the mean number of alleles per locus (A) was approximately three times higher for the anadromous population (A = 13.89) compared to the freshwater population (A = 4.89) (Wilcoxon signed rank test: N = 9, z = −2.670, p = 0.008; Table 1). However, both populations did not differ significantly from Hardy-Weinberg equilibrium (both p ≥ 0.159; Table 1) and inbreeding coefficient values were close to zero in both populations (freshwater: F_IS_ = 0.033; anadromous: F_IS_ = 0.035; Table 1), suggesting random mating patterns under natural conditions.

**Table 1:** **Population structure analysis**

**Population**	**N**	**Loci**	**A**	**H**_**e**_	**H**_**o**_	**F**_**IS**_	**p**_**HWE**_	χ^**2**^_**HWE**_	**df**_**HWE**_
freshwater	17	9	4.89	0.6686	0.6471	0.033	0.559	16.479	18
anadromous	17	9	13.89	0.9063	0.8758	0.035	0.159	23.868	18

Shown is a comparison of the variation of nine microsatellite markers based on 34 randomly chosen unrelated individuals (17 F1 fish from the freshwater and 17 F1 fish from the anadromous stickleback population) (see [41,69], and Additional file 1 for further details]. N: number of individuals typed, A: mean number of alleles per locus, H_e_: expected heterozygosity, H_o_: observed heterozygosity, F_IS_: inbreeding coefficient, and results of chi-square tests for deviation from expected proportions under Hardy-Weinberg equilibrium (HWE) (given are p-values, χ^2^ and degrees of freedom, df).

### *In vitro* fertilization trials

**Figure 1:**
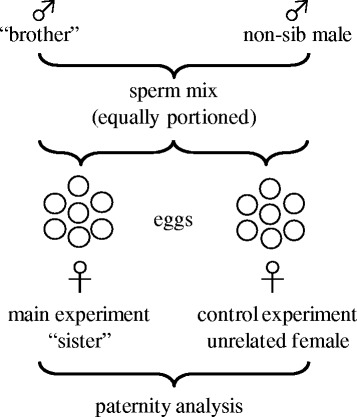
Experimental design. In each trial two males and two females were used. In the main experiment one male (“brother”) was related to the female (“sister”), whereas in the control experiment both males (“brother” and non-sib male) were unrelated to the female (unrelated female) (the number of fertilization trials consisting of two sub-trials each were N_freshwater_ = 17; N_anadromous_ = 22).

For both populations, *in vitro* fertilization trials (N_freshwater_ = 17, N_anadromous_ = 22) were conducted following an identical protocol. In detail, an *in vitro* fertilization trial consisted of two sub-trials in which sperm from the same two males were allowed to compete against each other for egg fertilization. For each sub-trial a different egg-donating female was used (see Figure 1). In one sub-trial (called main experiment) one of the two males was the female’s full-sib brother. In the other sub-trial (called control experiment) the female was unrelated to both males (see also Figure 1). Males are therefore referred to as “brother” (between quotation marks as brother is only valid for the main experiment) and non-sib male from now on.

Prior to *in vitro* fertilization trials randomly chosen males were isolated in single tanks (30 cm length × 20 cm width × 20 cm height) under summer conditions (see above), each equipped with a sand-filled Petri dish (Ø 9 cm) and 2 g of nest-building material (*Vesicularia dubyana*). Females used in the main experiments originated from the same laboratory-bred F1 generations (see above) whereas those used in the control experiments were wild-caught. This applies to the experiments with both the anadromous and the freshwater population. Wild-caught fish were trapped on March 27, 2008 (freshwater) and on April 3, 2009 (anadromous). All wild-caught fish were kept in mixed-sex groups of about 400–500 individuals in large outdoor tanks (750 l) with a constant supply of tap-water (3 l min^−1^) and were daily fed with *Chironomus* spec. In both stickleback populations, reproduction takes place one year after hatching. Accordingly, the frequency distribution of fish lengths is single-peaked (MM unpublished observation) revealing that wild-caught females used in the control experiment were of the same age as F1 individuals.

Only sperm of size-matched (± 2 mm), nest-holding males were allowed to compete against each other for egg fertilization (for details concerning the experimental design see Figure 1). First, one male of the pair was killed quickly by decapitation in order to dissect the testes as sperm stripping is not possible in sticklebacks (the only exception in [70]). Directly before decapitation fish were anesthetized with a blow to the head, which is the quickest method (see also [71]). After dissection, testes were separately stored in 200 μl of a non-activating medium (for mixture see [72]) for later *in vitro* fertilization (sperm from the left testis) and sperm morphology determination (sperm from the right testis). The same was done for the second male. To avoid sequence effects “brother” and non-sib males were killed in random order. Directly thereafter sperm number was determined for both males as described in Mehlis & Bakker [68] to ensure that equal proportions of males’ sperm (125,000 sperm per egg and male) were used during the fertilization process. By keeping the sperm number constant we controlled for a potential influence of sperm quantity as suggested by theory [73,74]. Immediately after both males’ testes had been dissected and sperm number had been quantified, one randomly chosen gravid female (either a “sister” of one of the males (main experiment) or a female unrelated to both males (control experiment), see Figure 1) was stripped and 50 eggs were counted and placed in a small glass Petri dish that already contained 1 ml of tap water. Using a pipette the sperm mixture (containing 125,000 sperm per egg of the “brother” and 125,000 sperm per egg of the non-sib male) was distributed over the eggs. The same was done with the other females’ eggs. Again, “sisters” and unrelated females were stripped in random order to avoid sequence effects.

One hour later, the fertilization process was stopped by adding sparkling table water over the clutches; a method which has been shown not to harm the eggs but to quickly kill the sperm [75]. Eggs were placed in a small aerated container (1 liter) and 24 h later fertilization rate was checked [76] using a binocular (Leica S8AP0), before all eggs were stored in 99.8% ethanol at −18°C for subsequent paternity analyses. Shortly before storage, ten randomly chosen fertilized eggs were weighed to the nearest milligram to determine the average egg mass as an indicator of female quality [77]. Females that participated in an *in vitro* fertilization sub-trial were marked by cutting the tip of a spine before they were returned to their holding tank in order to avoid repeated use and thus pseudoreplication. The females’ spines and tissue samples from the pectoral fin of the males were separately stored in 99.8% ethanol at −18°C for subsequent paternity analyses (see below).

Sperm morphology variables (head length (including mid-piece) (hl) and tail length (tl)) were determined by scanning electron microscopy (see [51,68] for details). Sperm morphology variables were based on 10.56 ± 3.74 (mean ± SD) sperm per male for the freshwater population and on 20.57 ± 1.99 (mean ± SD) sperm per male for the anadromous population.

### Paternity analyses

Paternity analyses were done using four polymorphic microsatellite markers ([41,69], see also Additional file 2). DNA-samples of parents and eggs were extracted via Chelex (Bio-Rad, after [78]). The tailed primer method [79] was used for subsequent PCR (see Additional file 3 for details) and PCR-products were run on a CEQ 8800 Genetic Analysis System (Beckman Coulter) and analyzed via GenomeLabTM GeXP (version10.2). For both sub-trials (i.e. main as well as the control experiment) sub-samples of 30 eggs were genotyped. On average 29.06 ± 1.59 (mean ± SD) eggs per sub-trial could be successfully assigned to one father for the freshwater population and 28.91 ± 2.37 (mean ± SD) for the anadromous population, respectively. In total 2260 eggs were genotyped (freshwater: 988 eggs; anadromous: 1272 eggs).

### Statistical analyses

Non-parametric statistics were used as data significantly deviated from normal distribution according to Kolmogorov-Smirnov tests with Lilliefors correction. Data were analyzed in SPSS 15.0. Test probabilities are two-tailed throughout. Linear mixed-effect models were fitted using the ‘lme’ function in the “nlme” library of the R 3.0.2 statistical package. The differences in fertilization success (“brother” minus non-sib male) were calculated for each population and used as dependent variable, the sub-trial (main or control experiment) was included as explanatory variable and trial number was defined as random factor and never removed to control for the paired study design.

An additional linear mixed-effect model was run to elucidate whether any sperm morphology trait was related to fertilization success. Therefore, differences in fertilization success (“brother” minus non-sib male) were again used as dependent variable and averaged values for males’ sperm tail length respectively the sperm head to tail length ratio (hl/tl, see [80]) were separately included as explanatory variable. Moreover, two different males as well as two females were used for one *in vitro* fertilization trial. Thus, males’ body size and females’ egg mass were included as explanatory variables and never removed to control for potential differences in males’ phenotype and females’ egg quality. Additionally to the trial number, the study population was defined as random factor. Both random factors were left in the model to control for the paired study design as well as for potential population differences (see Additional file 4 for an overview of all fitted models).

Tests of significance were based on likelihood-ratio tests and in all models explanatory variables were stepwise removed in the order of statistical relevance. The residuals of the best explaining models did not significantly deviate from normal distributions according to Kolmogorov-Smirnov tests.

## Results

**Figure 2:**
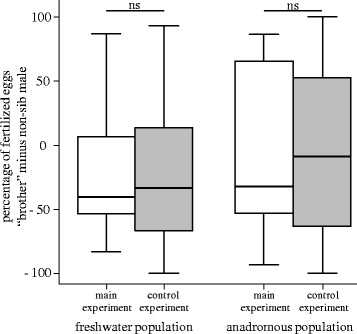
Results of the paternity analyses. Percentage of fertilized eggs (“brother” minus non-sib male) for the main and the control experiment both for the freshwater (N_freshwater_ = 17) as well as the anadromous population (N_anadromous_ = 22) plotted as median, quartiles and range. Negative values indicate that the non-sib male won during sperm competition trials. ns, not significant.

**Figure 3:**
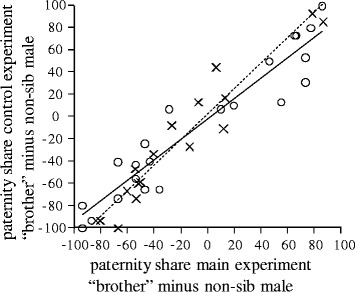
Relationship between the paternity share in the main experiment and in the control experiment. Shown are the data points and regression lines for both the freshwater (N = 17, crosses, broken line indicates the least-square linear regression line) and for the anadromous (N = 22, circles, solid line indicates the least-square linear regression line) population. For statistics see text.

The proportion of fertilized eggs did not differ significantly between the main and the control experiment (Wilcoxon signed rank test: N_freshwater_ = 17, z = −1.436, p = 0.151; N_anadromous_ = 22, z = −0.714, p = 0.475). In addition, the relative number of fertilized eggs (“brother” minus non-sib male) did not differ significantly between main and control experiment; neither in the freshwater population (“lme”, N_freshwater_ = 17, χ^2^ = 0.148, p = 0.700; Figure 2) nor in the anadromous population (“lme”, N_anadromous_ = 22, χ^2^ = 0.229, p = 0.632; Figure 2), indicating that no postcopulatory inbreeding avoidance mechanism exists in both populations. However, males that were successful in the control experiment were also the winner in the main experiment and *vice versa* (Pearson correlation: N_freshwater_ = 17, r_P_ = 0.961, p < 0.001; N_anadromous_ = 22, r_P_ = 0.950, p < 0.001; Figure 3).

**Figure 4:**
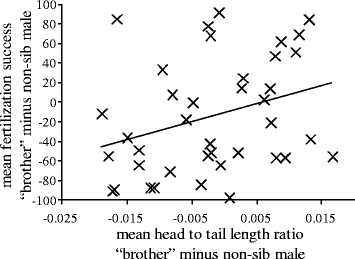
Sperm morphology in relation to fertilization success. Shown are the differences in fertilization success and the differences in sperm head to tail length ratio (“brother” minus non-sib male). Data for the main and the control experiment were pooled N = 39. The line indicates the least-square linear regression line. For statistics see text.

As in both populations postcopulatory inbreeding avoidance was absent, data were pooled for further analyses. The results showed that competitive fertilization success was significantly predicted by sperm quality (tail length: “lme”, N = 39, χ^2^ = 4.909, p = 0.027; head to tail length ratio: “lme”, N = 39, χ^2^ = 4.398, p = 0.036; Table 2). Fertilization success was significantly negatively correlated with tail length (Spearman rank correlation: N = 39, r_S_ = −0.349, p = 0.030) and significantly positively correlated with head to tail length ratio (Spearman rank correlation: N = 39, r_S_ = 0.319, p = 0.048; Figure 4).

**Table 2:** **Sperm morphology traits in relation to fertilization success**

**Dependent variable**		
**Competitive fertilization success**		
**Explanatory variable**	**χ** ^**2**^	**p-value**
Tail length	4.909	0.027
Head to tail length ratio	4.398	0.036

Both sperm tail length and head to tail length ratio significantly explain fertilization success in the *in vitro* sperm competition trials with fish from a freshwater and an anadromous stickleback population. Given are p-values and χ^2^. See text for details.

## Discussion

Both in a genetically heterogeneous, anadromous stickleback population and in a genetically impoverished, freshwater stickleback population, we found no evidence for a postcopulatory inbreeding avoidance mechanism. Males related and unrelated to the female, had on average equal paternity chances in sperm competition experiments. Paternity was related to the size of a male’s sperm instead.

Previous studies on sticklebacks of the present anadromous population have shown a precopulatory inbreeding avoidance mechanism as females preferred to mate with unrelated males [48,49]. In addition, males from the anadromous population released fewer sperm during incestuous matings (MM, LK Hilke and TCMB unpublished data). Hence, at least in the anadromous population both sexes seem to avoid the severe consequences of inbreeding at the precopulatory stage, which may explain the lack of a postcopulatory inbreeding avoidance mechanism as observed in the present study (see [81] for a comparable result). Whether this also applies to individuals originating from the small, genetically less diverse freshwater population is unknown. Indeed, in small populations the risk to mate with a partner by chance that carries genes identical by descent is obviously higher. By using *in vitro* fertilization techniques we avoided confounding influences of precopulatory differences in behavior and were thus able to investigate the existence of an inbreeding avoidance mechanism exclusively based on the postcopulatory level. Irrespective of population size and thus contrary to our expectations, we did not find any evidence for a postcopulatory inbreeding avoidance mechanism in the present study. However, previous studies showed that in guppies (*Poecilia reticulata*) and in lake trouts (*Salvelinus namaycush*) sperm swam faster in ovarian fluid of unrelated females indicating fitness benefits (see [82,83]). Nevertheless, a postcopulatory inbreeding avoidance exclusively based on sperm-egg interactions seems to be absent in sticklebacks.

An individual’s ability to select a mating partner that carries good or compatible genes could lead to indirect genetic benefits [84-86]. In detail, selection for “good genes” (additive genetic effects for increased survival) leads to directional selection, i.e. each female within a population should mate with males that carry these good genes. Selection for compatible genes (non-additive genetic variation) does not result in directional selection. For example, a high degree of relatedness between mating partners might represent one reason of genetic incompatibility [87,88]. In the present study the success of a male in the competitive fertilization trials was exclusively based on sperm quality (in terms of sperm morphology as we were not able to measure sperm velocity) in both populations. Hence, the results suggest a directional selection for a “superior” male phenotype independent of genetic incompatibility (see [27] for a comparable result in mallards). A recent study by Eizaguirre et al. [89] supported the good gene hypothesis of sexual selection in sticklebacks. In semi-natural enclosures, females preferred to mate with males with a specific MHC-haplotype, which was related to indirect fitness benefits as these males were larger and had a higher resistance to a common parasite (*Gyrodactylus* spec.) [89].

Between-male variation in stickleback sperm design (see also [67]) mainly consists of variation in sperm tail length, as it accounts for up to 90% of the total sperm length. Sperm with a longer tail (i.e. smaller head to tail length ratio, defined after [80]) swim in a more linear path and sperm that swim in a linear path were shown to swim faster [90], suggesting that these sperm might encounter an unfertilized egg more rapidly (see also [91]). In sperm competition, one would thus expect an advantage for longer sperm. Counterintuitively, in the present study relatively shorter sperm (and sperm with a relatively large head to tail length ratio) won the race for egg fertilization during competitive *in vitro* fertilization trials. A recent *in vitro* study by Bakker et al. [67] provides a convincing explanation. By experimentally manipulating fertilization duration Bakker et al. [67] showed that sperm with longer tails (i.e. relatively small head to tail length ratio) fertilize faster when the fertilization process was stopped 60 seconds after the start of the sperm release. This resulted in a positive correlation between sperm size and fertilization success whereas a negative correlation was found when the fertilization process was stopped after 600 seconds [67]. Thus, sperm size seems to be traded off against sperm longevity in our study species (see [67] for details). Additionally, in sticklebacks, complete clutch fertilization takes atypically long for an externally fertilizing species (up to 10 min, [75]) and the sperm motility period is prolonged by the presence of ovarian fluid (up to several hours, [92]).

To ensure high fertilization rates under laboratory conditions (on average 98% for the anadromous population and 96% for the freshwater population) we decided to stop the fertilization process after 60 minutes in the present study. This might account for the observed negative correlation between sperm size and fertilization success (see also [67] for details). As mentioned above, the relationship between sperm size and fertilization success is ambiguous, showing no general pattern: negative (e.g. [61,62]), positive (e.g. [63,64]) or absent (e.g. [65,66]). Generally, this topic is controversial and even within the same species the relationship between sperm morphology traits and sperm velocity in particular seems to be dynamic and influenced by many factors. Rick et al. [93], for example, showed that in sticklebacks, sperm velocity is negatively affected by increased levels of ambient UV light whereas sperm morphology traits remain unaffected.

## Conclusion

There is no evidence for a postcopulatory inbreeding avoidance mechanism in our study species, irrespective of population size. Instead, sperm quality traits predicted paternity success during competitive fertilization trials, indicating that this trait is under strong sexual selection, presumably forced by the high risk of sperm competition under natural conditions.

## Additional files

Additional file 1:
**List and PCR conditions of the microsatellite markers used for population structure analysis.**
Additional file 2:
**Details of the microsatellite markers used for paternity analyses.**
Additional file 3:
**PCR conditions of the microsatellite markers used for paternity analyses.**
Additional file 4:
**Overview of all fitted linear mixed-effect models.**
