# Supplementary material for: Sperm quality but not relatedness predicts sperm competition success in threespine sticklebacks (Gasterosteus aculeatus)
Source: BMC Evol Biol. 2015 Apr 26;15:74. doi: 10.1186/s12862-015-0353-x (PMC4415302; doi:10.1186/s12862-015-0353-x)
Supplement: Additional file 1: — List and PCR conditions of the microsatellite markers used for population structure analysis. [file 12862_2015_353_MOESM1_ESM.docx]

**Additional file 1 List and PCR conditions of the nine microsatellite markers [1, 2] used in the population structure analysis.**

| Locus | GenBank  accession no. | dye label | tail | tail-sequence 5'- 3‘ |
| --- | --- | --- | --- | --- |
| GAC1097PBBE | AJ010352 | D2 (black) | M13 | TGTAAAACGACGGCCAGT |
| GAC1116PBBE | AJ010353 | D3 (green) | T7 | TAATACGACTCACTATAG |
| GAC1125PBBE | AJ010354 | D2 (black) | M13 | TGTAAAACGACGGCCAGT |
| GAC3133PBBE | AJ010356 | D4 (blue) | Sp6 | GATTTAGGTGACACTAT |
| GAC4170PBBE | AJ010357 | D4 (blue) | Sp6 | GATTTAGGTGACACTAT |
| GAC4174PBBE | AJ010358 | D3 (green) | T7 | TAATACGACTCACTATAG |
| GAC5196PBBE | AJ010359 | D3 (green) | T7 | TAATACGACTCACTATAG |
| GAC7010PBBE | AJ311863 | D2 (black) | M13 | TGTAAAACGACGGCCAGT |
| GAC7033PBBE | AJ010360 | D4 (blue) | M13 | TGTAAAACGACGGCCAGT |

| PCR program:  GAC1097PBBE, GAC1125PBBE, GAC4170PBBE, GAC5196PBBE | |  | PCR program:  GAC1116PBBE, GAC3133PBBE, GAC4174PBBE, GAC7010PBBE  GAC7033PBBE | |
| --- | --- | --- | --- | --- |
| preheating | 94 °C 15 min. |  | preheating | 94 °C 15 min. |
| 30 cycles |  |  | 30 cycles |  |
| denaturing | 94 °C 60 sec. |  | denaturing | 94 °C 60 sec. |
| annealing | 58 °C 45 sec. |  | annealing | 56 °C 45 sec. |
| elongating | 72 °C 60 sec. |  | elongating | 72 °C 60 sec. |
| 8 cycles |  |  | 8 cycles |  |
| denaturing | 94 °C 60 sec. |  | denaturing | 94 °C 60 sec. |
| annealing | 53 °C 45 sec. |  | annealing | 53 °C 45 sec. |
| elongating | 72 °C 60 sec. |  | elongating | 72 °C 60 sec. |
| final extension cycle | 72 °C 30 min. |  | final extension cycle | 72 °C 30 min. |

Tissue samples were extracted via Chelex [Bio-Rad, 3]. The tailed primer method [4] was used for subsequent PCR and PCR-products were run on a CEQ 8800 (Beckman Coulter) and analyzed via GenomeLabTM GeXP (version10.2).

To estimate genetic diversity 17 unrelated F1-descendants of wild-caught fish of each of the two populations used in the fertilization trials were genotyped for the nine microsatellite loci given above. Number of alleles (A), observed heterozygosity (H_o_), and Nei’s unbiased gene diversity [H_e_, 5] were calculated using the Microsatellite Toolkit for MS Excel [6]. The web-based version of Genepop 4.2 [7, http://genepop.curtin.edu.au] was used to calculate the inbreeding coefficient F_IS_ [after 8] and deviations from Hardy-Weinberg equilibrium [9, Markov chain Monte Carlo simulation with dememorization 5000, batches 500 and iterations per batch 5000).

1. Heckel G, Zbinden M, Mazzi D, Kohler A, Reckeweg G, Bakker TCM, Largiadèr CR: **Microsatellite markers for the three-spined stickleback (*Gasterosteus aculeatus* L.) and their applicability in a freshwater and an anadromous population.** *Conserv Genet* 2002, **3**:79-81.

2. Largiadèr CR, Fries V, Kobler B, Bakker TCM: **Isolation and characterization of microsatellite loci from the three-spined stickleback (*Gasterosteus aculeatus* L.)**. *Mol Ecol* 1999, **8**:342-344.

3. Estoup A, Largiadèr CR, Perrot E, Chourrout D: **Rapid one-tube DNA extraction for reliable PCR detection of fish polymorphic markers and transgenes.** *Mol Mar Biol Biotechnol* 1996, **5**:295-298.

4. Schuelke M: **An economic method for the fluorescent labeling of PCR fragments.** *Nat Biotechnol* 2000, **18**:233-234.

5. Nei M: *Molecular evolutionary genetics.* New York: Columbia University Press; 1987.

6. Park R: **Trypanotolerance in West African cattle and the population genetic effects of selection.** *PhD thesis.* University of Dublin, 2001.

7. Raymond M, Rousset F: **GENEPOP (version 1.2): population genetics software for exact tests and ecumenicism.** *J Hered* 1995, **86**:248-249.

8. Weir BS, Cockerham CC: **Estimating F-statistics for the analysis of population-structure.** *Evolution* 1984, **38**:1358-1370.

9. Guo SW, Thompson EA: **Performing the exact test of Hardy-Weinberg proportion for multiple alleles.** *Biometrics* 1992, **48**:361-372.
