# Supplementary material for: Sperm quality but not relatedness predicts sperm competition success in threespine sticklebacks (Gasterosteus aculeatus)
Source: BMC Evol Biol. 2015 Apr 26;15:74. doi: 10.1186/s12862-015-0353-x (PMC4415302; doi:10.1186/s12862-015-0353-x)
Supplement: Additional file 2: — Details of the microsatellite markers used for paternity analyses. [file 12862_2015_353_MOESM2_ESM.docx]

**Additional file 2 Details of the four microsatellite markers [1, 2] that were used for the paternity analyses.**

| Locus | GenBank  accession no. | dye label | tail | tail-sequence 5'- 3‘ |
| --- | --- | --- | --- | --- |
| GAC1116PBBE | AJ010353 | D3 (green) | T7 | TAATACGACTCACTATAG |
| GAC2142PBBE | AJ311857 | D4 (blue) | Sp6 | GATTTAGGTGACACTAT |
| GAC4174PBBE | AJ010358 | D3 (green) | T7 | TAATACGACTCACTATAG |
| GAC7033PBBE | AJ010360 | D4 (blue) | M13 | TGTAAAACGACGGCCAGT |

For both populations all putative fathers and mothers were genotyped first. Thereafter, it was separately decided which microsatellite marker was meaningful and used for egg-genotyping later on. On average, each egg was genotyped with two microsatellites markers (freshwater population: 2.19 ± 0.72 (mean ± SD); anadromous population: 2.02 ± 0.55 (mean ± SD)) of which at least one (freshwater population: 1.25 ± 0.45 (mean ± SD); anadromous population: 1.54 ± 0.59 (mean ± SD)) was 100 % informative.

To successfully assign fatherhood, fourteen eggs, their mothers and putative fathers were additionally genotyped with four additional microsatellite markers [see 2].

| Locus | GenBank  accession no. | dye label | tail | tail-sequence 5'- 3‘ |
| --- | --- | --- | --- | --- |
| GAC1097PBBE | AJ010352 | D2 (black) | M13 | TGTAAAACGACGGCCAGT |
| GAC1125PBBE | AJ010354 | D2 (black) | M13 | TGTAAAACGACGGCCAGT |
| GAC4170PBBE | AJ010357 | D4 (blue) | Sp6 | GATTTAGGTGACACTAT |
| GAC5196PBBE | AJ010359 | D3 (green) | T7 | TAATACGACTCACTATAG |

1. Heckel G, Zbinden M, Mazzi D, Kohler A, Reckeweg G, Bakker TCM, Largiadèr CR: **Microsatellite markers for the three-spined stickleback (*Gasterosteus aculeatus* L.) and their applicability in a freshwater and an anadromous population.** *Conserv Genet* 2002, **3**:79-81.

2. Largiadèr CR, Fries V, Kobler B, Bakker TCM: **Isolation and characterization of microsatellite loci from the three-spined stickleback (*Gasterosteus aculeatus* L.)**. *Mol Ecol* 1999, **8**:342-344.
