# Supplementary material for: Sperm quality but not relatedness predicts sperm competition success in threespine sticklebacks (Gasterosteus aculeatus)
Source: BMC Evol Biol. 2015 Apr 26;15:74. doi: 10.1186/s12862-015-0353-x (PMC4415302; doi:10.1186/s12862-015-0353-x)
Supplement: Additional file 3: — PCR conditions of the microsatellite markers used for paternity analyses. [file 12862_2015_353_MOESM3_ESM.docx]

**Additional file 3 PCR conditions of the microsatellite markers used for paternity analyses.**

| PCR program:  GAC1116PBBE, GAC2142PBBE, GAC4174PBBE, GAC7033PBBE | | | |
| --- | --- | --- | --- |
| preheating | 94 °C 15 min. | | |
| 40 cycles |  | | |
| denaturing | 94 °C 30 sec. | | |
| annealing | 60 °C 30 sec. | | |
| elongating | 72 °C 60 sec. | | |
| final extension cycle | 72 °C 30 min. | | |
|  | |  |  |
| PCR program:  GAC1097PBBE, GAC1125PBBE, GAC4170PBBE, GAC5196PBBE | | | |
| preheating | 94 °C 15 min. | | |
| 30 cycles |  | | |
| denaturing | 94 °C 60 sec. | | |
| annealing | 58 °C 45 sec. | | |
| elongating | 72 °C 60 sec. | | |
| 8 cycles |  | | |
| denaturing | 94 °C 60 sec. | | |
| annealing | 53 °C 45 sec. | | |
| elongating | 72 °C 60 sec. | | |
| final extension cycle | 72 °C 30 min. | | |
