# Supplementary material for: Sperm quality but not relatedness predicts sperm competition success in threespine sticklebacks (Gasterosteus aculeatus)
Source: BMC Evol Biol. 2015 Apr 26;15:74. doi: 10.1186/s12862-015-0353-x (PMC4415302; doi:10.1186/s12862-015-0353-x)
Supplement: Additional file 4: — Overview of all fitted linear mixed-effect models. [file 12862_2015_353_MOESM4_ESM.docx]

**Additional file 4 Overview of all fitted linear mixed-effect models.**

| model-no. | dependent variable | explanatory variable | random factor |
| --- | --- | --- | --- |
| 1. a) | percentage of fertilized eggs  (freshwater population) | sub-trials (main or control experiment) | trial number |
| b) | percentage of fertilized eggs  (anadromous population) | sub-trials (main or control experiment) | trial number |
| 2. a) | percentage of fertilized eggs | tail length  body size (male)  egg mass | population & trial number |
| b) | percentage of fertilized eggs | head to tail length ratio  body size (male)  egg mass | population & trial number |

In all models, explanatory variables were stepwise removed in the order of statistical relevance. In model-no. 2. a) and 2. b) body size (male) and egg mass were left in the model to control for potential differences in males’ phenotype and females’ egg quality. In addition, in all models random factors (trial number and population) were never removed to control for the paired study design and potential population differences, respectively.
